# Patients’ gut feelings seem useful in primary care professionals’ decision making

**DOI:** 10.1186/s12875-022-01794-9

**Published:** 2022-07-20

**Authors:** C. F. Stolper, M. W. J. van de Wiel, M. A. van Bokhoven, G. J. Dinant, P. Van Royen

**Affiliations:** 1 CAPHRI School for Public Health and Primary Care, University of Maastricht, Maastricht, The Netherlands; 2 Faculty of Medicine and Health Sciences, Department of Family Medicine and Population Health, University of Antwerp, Antwerp, Belgium; 3 Department of Work and Social Psychology, Faculty of Psychology and Neuroscience, Maastricht University, Maastricht, The Netherlands

**Keywords:** Patient intuition, Patients’ gut feelings, Primary care, Clinical reasoning, General practitioners, Family practice, Family physicians

## Abstract

**Background:**

Family physicians’ diagnostic gut feelings have proved to be valuable. But what about patients’ gut feelings? Research has shown that patients’ gut feelings may contribute to their physicians’ clinical reasoning. Dutch medical tribunals consider patients’ worry useful for doctors’ diagnostic process. However, how general practitioners and other primary care professionals recognize gut feelings of patients and deal with them in their decision making is yet unclear. We aim to explore how primary care professionals perceive patients’ gut feelings and use this information in their decision-making.

**Methods:**

We interviewed 30 Dutch and Belgian primary care professionals, exploring how they recognize and value patients’ gut feelings. We coded all interviews using a descriptive content analysis in an iterative process. Data sufficiency was achieved.

**Results:**

Primary care professionals acknowledged gut feelings in their patients, and most participants found them a useful source of information. Patients’ gut feelings might alert them to possible hidden problems and might provide quicker insight into patients’ perceptions. Primary care professionals listed a whole series of wordings relating to trusting or distrusting the situation or to any changes in normal patterns. A patient’s gut feeling was often a reason for the professionals to explore patients’ worries and to reconsider their own clinical reasoning.

**Conclusions:**

Primary care professionals regularly considered patients’ gut feelings useful, as they might contribute to their clinical reasoning and to a deeper understanding of the patient’s problem. The next step could be to ask patients themselves about their gut feelings and explore their diagnostic value.

## Background

The role of gut feelings in the clinical reasoning of general practitioners (GPs) has been thoroughly examined and proved to be substantial [1–5]. An initial exploratory study of this topic among hospital specialists found comparable results [6]. The concept of gut feelings among GPs is related to the general concept of intuition, which has been described as ‘automatic knowing’ [7–9]. Physicians’ gut feelings, consisting of a sense of reassurance and a sense of alarm, as well as various determinants thereof, is well-defined and a key feature of GPs’ clinical reasoning in Europe [10–13]. (www.gutfeelingsingeneralpractice.eu) In different languages, physicians use specific expressions to voice their gut feelings [12].

Surprisingly, gut feelings of patients have been less studied until now [14]. Only a few studies have explored the credibility and significance of patient intuition [15–19]. A qualitative study showed that patients’ gut feeling about having cancer could be an important reason for further diagnostics [16]. A prospective, observational study found that parents’ feeling that ‘this illness is different from previous illnesses’ had a high positive predictive value (LR + 14,4) for serious infections among children [15, 18]. Triage nurses were able to quantify patients’ degree of worry [17]. These studies have shown that patients’ gut feelings exist, might have a diagnostic value in primary care and might be measurable. Additionally, patients are usually most familiar with their own body and may use this experiential knowledge in their decision to consult a physician. The well-known saying ‘mother knows best’ indicates the value of mothers’ assessment of their child’s health Remarkably, medical disciplinary tribunals in the Netherlands state that patients’ or relatives’ worries should be taken into account in the diagnostic process, which should result in reviewing their clinical reasoning [20].

It is, however, still unknown how primary care professionals such as GPs, practice and triage nurses, and desk staff recognize and value patients’ gut feelings and how patients’ gut feelings influence the professionals’ clinical reasoning and actions.

## Methods

Our aim was to clarify how primary care professionals recognize and value patients’ gut feelings and how patients’ gut feelings influence the professionals’ clinical reasoning and actions.

### Participants

We opted for purposeful sampling to maximize the exploration of different perspectives. Therefore, we recruited Dutch and Belgian primary care professionals both working in daily practices and out-of-hours GP services, ranging from those who have the first contact with patients up to GPs at the end of the primary care chain. We interviewed GPs, practice nurses working in GP practice, as well as triage nurses or desk staff working in out-of-hours GP services. Practice nurses and triage nurses assess the level of urgency of a patient’s request for consulting a GP. They decide whether and how promptly a patient will get an appointment with the GP, whereas the desk staff answers the phones, and assess whether a home visit, consultation or telephone response by the GP is appropriate.

We first mailed or phoned potential participants and asked them for permission to interview them about their views on patients’ gut feelings. Some of the participants had been known to us; others were found via snowball sampling. Next, we planned the interviews face-to-face or by phone, for logistic reasons. We (CFS and PVR) interviewed 30 experienced primary care professionals: 8 Dutch (4 female) and 5 Belgian GPs (all male), 13 Dutch nurse practitioners and triage nurses (all female), and 4 Belgian desk staff workers (all female). Some of the nurse practitioners and triage nurses preferred to be interviewed in small groups of two or three persons.

### Interviews and analysis

We developed a semi-structured interview guide to examine the research questions asking participants whether they recognized patients’ gut feelings and how, what role these gut feelings played in their clinical reasoning and to what kind of actions this led. Audio recordings of the interviews were transcribed verbatim and in an inductive way analysed using a descriptive content analysis approach [21, 22]. The coding was done by CFS and checked by PVR. We composed a coding book in an iterative process starting from the main themes addressed in the interviews and including emergent themes, until data sufficiency was achieved. All research team members were engaged in discussion on the codes, concepts, and themes. We have found the following five themes: recognition and difference, wordings and expressions, perceived value, acting upon gut feelings and proactive use. Below, we provide summaries of the themes, illustrated by quotes from participants.

## Results

### Recognition and difference

Almost all interviewees acknowledged the existence of gut feelings in patients, recognizing the feelings as a sense of alarm or a sense of reassurance. The three groups, GPs, nurse practitioners and triage nurses, did not essentially differ concerning their view on the topic. Some GPs considered patients’ gut feelings comparable to their own gut feelings, but in their view patients’ gut feelings were based on much less medical knowledge. A Dutch GP said: *Of course, while we doctors work by using a lot of knowledge and a lot of experience, patients really only use experience.* Primary care professionals noted that the expressions patients used to convey their gut feelings differed from the medical jargon professionals use (sense of reassurance/sense of alarm; ‘*pluis/niet-pluis’ in Dutch*).

### Wordings and expressions used by patients

Almost all participants recognized gut feelings of patients by verbal cues, and by non-verbal, paralinguistic cues. Primary care professionals reported that patients’ expressions of gut feelings were of two types, i.e., relating to trusting or distrusting the situation or on any changes in normal, familiar patterns. Most of the verbal cues given by patients or parents of ill children that the professionals mentioned in the interviews concerned a sense of alarm formulated as *I do not trust this; I feel something is not right; I ‘m worried; I’m frightened that it’s something bad; something must be done as I can’t go on like this; it doesn’t fit; I feel different from normal; this is not how I am normally; it’s so strange; I’ve never seen my child like this before; this is not like my child.* A sense of reassurance was much less often mentioned: *I’m not worried; I think it’ll be all right; I feel okay about it; not that I’m worried, but can you give me something for it?; this is normal for me.*

The non-verbal cues concerned the sound of patients’ voice: *You sometimes just hear it in their voice, rather than in the words they use (*Belgian triage nurse*). ‘But I still wanted to phone’, she said to me. In terms of triage, I couldn’t find anything alarming* [in the story about her ill child]*. Then I said, ‘But I sense that you’re worried.’ ‘I am’, she said, and I could hear the relief in her voice: she understands me. It might be nothing, but the mother had a sense of alarm* (Dutch triage nurse). Patients’ body language might also make primary care professionals pay attention to their patients’ gut feelings *because of people’s body language, without them using specific words, but because they behave differently and tell things in a different way from how we know them* (Belgian GP).

Although patients might say that they are not really worried, they sometimes still want to be reassured. According to primary care professionals, patients might say: *I don’t think anything’s really wrong, but I just wanted to phone you* (Dutch triage nurse) or *As long as I know it’s okay and get that confirmed by you* (Dutch GP).

Most Dutch and Belgian participants mentioned the same wordings and expressions being used by patients to voice their gut feelings. According to some Belgian primary care professionals, their patients are cautious about communicating their gut feelings with them at the beginning of a consultation: *If they themselves think there’s something wrong, but they won’t say that to me. They then start to talk about things like how high the fever is, how much coughing there is. Those kinds of things.* (Belgian GP).

### Perceived value of patients’ gut feelings

Several factors played a role in primary care professionals’ assessment of the value of gut feelings of patients or their relatives. If the professional knows a patient, they can use their knowledge of the patient, such as medical history, behaviour and health anxiety. *As a care provider you then address and assess very different things; you notice them, often subconsciously, like, this is not as usual, there’s something else going on here. Just based on people’s body language, without them using specific words, but because they behave differently* (Belgian GP). But if the professionals do not know the patient, they miss a frame of reference. *‘It’s more difficult to assess whether someone at that moment is more agitated or anxious in the way they express themselves than usual* (Belgian GP). They do weigh their patients’ gut feelings against their own judgement: *I get patients coming to see me, who* [for instance, CFS] *had a back pain since yesterday and then say doctor I’m worried about it because of such and such. I will then not so much get a sense of alarm as when I have doubts about it myself. It’s really a combination of various factors* (Dutch GP).

In the way primary care professionals voiced their own gut feelings they sometimes adopt a patient’s gut feeling: *If a parent indicates that there’s something seriously wrong, that may give you a sense of alarm yourself, especially with young children* (Belgian GP). *Just yesterday I had an example, where this patient, a baby, because of the parents’ worries, which they transfer to you, was referred to the pediatrician anyway. And I definitely take that aspect into consideration* (Dutch GP). *Sometimes, when she also becomes worried by the patient’s concern, the practice nurse puts them* [the patient] *straight through to me,* (Dutch GP).

Primary care professionals paid extra attention to gut feelings of parents coming in with a sick child. *I do tend to be on the alert particularly with parents and children. Especially since children can’t express things very clearly, so I must try to get a picture of the child’s state through the parents* (Dutch triage nurse).

Pitfalls in the assessment of patients’ gut feelings were patients who played down their complaints or who exaggerated them, being over-anxious. *They want to consult you about it, in the hope that we will tell them it’s nothing, but actually they sometimes know that it might very well be something serious* (Dutch nurse-practitioner). *It’s particularly those people who are very easily reassured by us, where you sometimes get this sense of there’s something else behind this, so that you think well, it’s a bit unusual, this story* (Dutch GP).

### Acting upon a patient’s gut feeling

A patient’ gut feeling was often a reason for primary care professionals to go on asking questions to explore why patients were so worried and sometimes to reconsider their diagnostic hypotheses and management decisions: *So I then think, right, have I now really asked all the questions and do I know all now? Could there be something underlying or whatever? I get the feeling that they haven’t told me everything* (Dutch nurse-practitioner).

In some cases, the professional mentioned that they had to give the patient more information to reassure them: *For instance, it may be that they have too little knowledge. So, they become worried if their child has a temperature of 40°. You would first have to explain, like, ‘Well this temperature in itself is not the main problem, it’s more the general condition'. So yeah, knowledge comes into it, too. You have to provide that first* (Dutch nurse-practitioner).

According to the work protocols of the Dutch out-of-hours GP services, triage nurses have to record patients’ sense of alarm in the medical records and how they deal with it. For a nurse practitioner or triage nurse, a patient’s gut feeling might be a reason to urgently discuss the case with a GP.

According to some GPs, a patient’s gut feeling could be an argument to prescribe an antibiotic sooner: *The way I see it, with someone who’s worried about their health, I’ll be a bit more positive as regards prescribing or not prescribing antibiotics than with someone who doesn’t so much express concern* (Dutch GP). Other GPs were cautious about letting their management be guided too much by patients’ gut feelings: *You have to be cautious about letting the patient’s worries influence your diagnostics and management too much, otherwise you’ll just keep prescribing antibiotics for instance* (Belgian GP).

### Pro-active use of patients’ gut feelings

Some primary care professionals explored a patient’s gut feeling at an early stage in the consultation, as this could make the contact easier and enlighten the conversation: It helps *to address that* [a gut feeling] *at a fairly early stage in the consultation, as it can sometimes be very illuminating, and can often lead to a more logical, natural course of the consultation than when it comes up at the end, as a kind of purely formal question when the consultation already appears to be complete* (Dutch GP).

Primary care professionals mentioned that they sometimes anticipated a patient’s possible gut feeling, staying at the safe side in their advice to come back if they do not feel well: *If you feel in your guts that it’s not okay, then… I tell them you should not just see how high the fever is, or how often little Johnny coughs, that’s not important. What matters is the general impression that something’s wrong now … 'when you feel you’re worried about it, then I want you to contact the practice again, to really come back here'* (Belgian GP). Some primary care professionals might check at the end of the consultation whether there were still hidden any gut feelings.

## Discussion

### Key findings of the study

The Dutch and Belgian primary care professionals interviewed indicated that they easily recognized patients’ gut feelings. Most of them considered patients’ gut feelings a useful source of information for the communication with these patients and for their clinical reasoning, particularly when they know the patient from their practice. The professionals were able to list wordings and expressions that patients use to voice their gut feelings, particularly regarding the sense of alarm. The phrases indicated that patients trust or distrust the situation or experience changes in normal, familiar patterns. Sometimes the patients’ voice or their body language put them on the track of a patient’s gut feelings. Primary care professionals mentioned that they first tried to explore why a patient is worried and discuss it. In this process of exploration, they weighed the value of the patients’ gut feelings against their contextual knowledge about the patient and own assessment of the situation. This might result in them reconsidering their diagnostic hypotheses and might influence management decisions. Some professionals explored a patient’s gut feeling at an early stage in the consultation to facilitate the communication with patients or advised them when closing the consultation to come back in case of a gut feeling.

### In comparison with literature

GPs’ gut feelings are based on medical knowledge, contextual knowledge about patients, and clinical expertise, and are a well-known and valuable part of GPs’ clinical reasoning [9, 12]. The sense of alarm and the sense of reassurance are related to possible health outcomes and might initiate specific clinical reasoning, diagnostics, and management [9]. The triage nurses, nurse-practitioners, and desk staff workers we interviewed appeared to be familiar with this medical concept, which is in line with other findings [23]. Primary care professionals clearly recognized patients’ gut feelings and were able to discuss them with patients, although patients used other words and expressions to voice their feelings than they would use themselves. Primary care professionals seemed to use their own gut feelings concept as a frame of reference because they distinguished a sense of alarm or a sense of reassurance when reporting the patients’ gut feelings [1]. This raises the question whether the patients’ concept of gut feelings, regarding their own health situation or that of relatives, differs from the professionals’ concept of gut feelings. An important difference is that patients have different and often less accurate knowledge about health and diseases than physicians. On the other hand, they may have more experiential knowledge regarding positive or negative changes in relation to their illness. Dutch-speaking primary care professionals use a specific expression to voice their gut feelings (‘pluis/niet-pluis’), which facilitates discussions among colleagues [1]. In our study, the professionals have not reported such specific medical expression used by patients, but nevertheless, they had no problem recognizing them. Continuity of care and contextual knowledge are key features of primary care and support the primary care professional when assessing the value of a patient’s gut feeling as well as their own gut feelings. The PPV of GPs’ gut feelings are moderate to good [4, 5, 24, 25], but the absolute numbers of diagnostic value may be less important than the alerting role of gut feelings, both from professionals and patients, in starting up or triggering the clinical reasoning process of primary care professionals.

### Strengths and limitations

This study is the first to explore the view of primary care professionals on the usefulness of patients’ gut feelings for their clinical reasoning. The transferability of the results of our study is high as we included professionals ranging from those who have the first contact with patients up to GPs at the end of the primary care chain, both those working in daily practice and those working in out-of-hours GP services. Additionally, we interviewed professionals from two different countries. We consider our results of much relevance to health care systems with a comparable strong organised primary care, such as in many European countries. Since most members of the research team are GPs themselves, the particular GP perspective may have caused us to overlook other relevant aspects. Therefore, the input of the cognitive psychologist in the team (MWJvdW) was important. Other limitations of this study were that we did not study the credibility of patients’ gut feelings, and to what extent the professionals’ prejudices regarding the patient might bias their attention and response to patients’ gut feelings.

## Conclusions

In conclusion, primary care professionals regularly considered patients’ gut feelings useful, as they might contribute to their clinical reasoning and to a deeper understanding of the patient’s problem. The next step in this research could be to examine patients’ gut feelings by asking them about their feelings and experiences. It would be interesting to explore their wordings and expressions, to see whether they trust their gut feelings, how they communicate them to primary care professionals and whether they feel taken seriously by the professionals. This knowledge will improve the professional’s recognition of a patient’s gut feeling and insight in its background. In a final step we might compose and validate a questionnaire enabling a further exploration of the diagnostic value of patients’ gut feelings.

## Abbreviations

GP: General practitioner
PPV: Positive predictive value
